# New observations on gametogenic development and reproductive experimental tools to support seed yield improvement in cowpea [*Vigna unguiculata* (L.) Walp.]

**DOI:** 10.1007/s00497-015-0273-3

**Published:** 2016-01-04

**Authors:** Rigel Salinas-Gamboa, Susan D. Johnson, Nidia Sánchez-León, Anna M. G. Koltunow, Jean-Philippe Vielle-Calzada

**Affiliations:** Grupo de Desarrollo Reproductivo y Apomixis, Laboratorio Nacional de Genómica para la Biodiversidad, CINVESTAV Irapuato, CP 36821 Guanajuato, Mexico; Agriculture Flagship, Commonwealth Scientific and Industrial Research Organization, Private Bag 2, Glen Osmond, SA 5064 Australia

**Keywords:** Megagametogenesis, Megasporogenesis, Microgametogenesis, Cowpea, Reproductive calendar, Whole-mount immunolocalization, Ovule

## Abstract

*****Key message***:**

Cowpea reproductive tools.

**Abstract:**

*Vigna unguiculata* L. Walp. (cowpea) is recognized as a major legume food crop in Africa, but seed yields remain low in most varieties adapted to local conditions. The development of hybrid cowpea seed that could be saved after each generation, enabling significant yield increases, will require manipulation of reproductive development from a sexual to an asexual mode. To develop new technologies that could support the biotechnological manipulation of reproductive development in cowpea, we examined gametogenesis and seed formation in two transformable, African-adapted, day-length-insensitive varieties. Here, we show that these two varieties exhibit distinct morphological and phenological traits but share a common developmental sequence in terms of ovule formation and gametogenesis. We present a reproductive calendar that allows prediction of male and female gametogenesis on the basis of sporophytic parameters related to floral bud size and reproductive organ development, determining that gametogenesis occurs more rapidly in the anther than in the ovule. We also show that the mode of megagametogenesis is of the Polygonum-type and not Oenothera-type, as previously reported. Finally, we developed a whole-mount immunolocalization protocol and applied it to detect meiotic proteins in the cowpea megaspore mother cell, opening opportunities for comparing the dynamics of protein localization during male and female meiosis, as well as other reproductive events in this emerging legume model system.

**Electronic supplementary material:**

The online version of this article (doi:10.1007/s00497-015-0273-3) contains supplementary material, which is available to authorized users.

## Introduction

Many of the poorest countries in the world derive 10–20 % of their total dietary protein from grain legumes, members of the *Fabaceae* (Akibode and Maredia, 2011). Cowpea, *Vignaunguiculata* (L.) Walp. is one of the eight grain legumes currently being targeted for yield and agronomic improvement by the Consultative Group for International Agricultural Research (CGIAR). Cowpea originated in Africa, which produces over 95 % of the 5.4 million tons of dried cowpeas produced globally, on an estimated 11.5 million hectares of land across a diverse range of farming systems, in the semiarid regions of West and Central Africa. Approximately 200 million individuals are estimated to consume the grain daily in West Africa as it provides a high source of protein (25 % by weight) and all plant organs are edible. Cowpea is an important component in increasing sustainable farming production by being drought resistant and having the ability to fix atmospheric nitrogen, contributing to reduced soil erosion.

Conventional cowpea breeding is currently underpinned by the use of molecular markers (Lucas et al. 2011, 2013), correlations between genetic and physical maps (Pottorff et al. 2014), and large-scale genomic sequencing (Andargie et al. 2011; Barrera-Figueroa et al. 2011; Huang et al. 2012). Transformation technologies have been established for cowpea and are being implemented to accelerate agronomic performance, with strong indications of success in the development of seed borer-resistant lines (Kang et al. 2014; Popelka et al. 2006). Despite advances in increasing cowpea yields and stress tolerance over the last two decades, many varieties are still low yielding and susceptible to a range of biotic and abiotic stresses (Lush and Evans 1981; Singh 2014; Huynh et al. 2015; Lucas et al. 2015).

The development of cowpea hybrids has the potential to increase crop yields by 30 % as suggested by published studies aimed at identifying levels of heterosis in progeny from crosses between inbred cowpea parents (Bhaskaraiah et al. 1980; Ortiz 1998; Ushakumari et al. 2010; Kumari et al. 2012). Hybrid seed production systems have not yet been developed in cowpea. Impediments include self-fertilizing hermaphrodite flowers, low outcrossing rates (<5 %; Timko and Singh 2008), and high flower drop rates (Wien and Summerfield 1980; Ehlers and Hall 1996; Ojehomon and Samyaolu 1970). In all current hybrid seed production systems, hybrid seeds need to be generated and purchased each growing season, impeding their purchase by poor farmers. The current inability to produce hybrid seeds relates to the plant’s sexual reproductive process. Meiosis and recombination during gamete formation and gamete fusion at fertilization alleviate heterosis, concurrently leading to trait segregation. Development of cowpea hybrids from which seeds could be saved by farmers would offer the potential to generate higher-yielding cowpea crops in an economical manner. The generation of such “self-reproducing” hybrids would require changing reproduction in the sexually reproducing hybrid to an asexual seed forming mode mimicking the events of apomixis to generate clonal seed where vigor was preserved. Recent studies have provided proof-of-concept of clonal seed generation, which might be applicable for the generation of self-reproducing cowpea hybrids (Marimuthu et al. 2011; Hand and Koltunow 2014; Gilbert 2015).

While general descriptions of reproductive development in members of the *Fabaceae* have been published from a physiological and developmental perspective (Brown 1917; Mitchel 1975; Rembert 1977; Albertsen and Palmer 1979; Kennell and Horner 1985; Moço and Mariath 2003; Soverna et al. 2003; Rodriguez-Riaños et al. 2006; Chehregani and Tanaomi 2010; Ghassempour et al. 2011), there is surprisingly scarce information on micro- and megagametogenesis in *Vigna*. An important prerequisite for developing robust hybrid seed production systems or attempting to alter reproduction in cowpea is a clear understanding of the temporal and spatial events of sexual male and female gametophyte formation in relation to flower morphology and supporting cytological tools to efficiently and accurately examine changes induced by hybridization, mutation, or transgenic approaches.

In this paper, we applied a range of cytological techniques to a pair of cultivated African cowpea varieties of known pedigree developed by the International Institute of Tropical Agriculture (Kang et al. 2014) and developed a reproductive calendar that allows prediction of male and female gametogenesis on the basis of sporophytic parameters such as floral bud size, gynoecium length, and integument growth. Although one flowers earlier than the other, both share a common developmental sequence in terms of ovule formation and gametogenesis. Contrary to previous reports, we show that the female gametophyte is not of the Oenothera -type but consistently develops from three mitotic nuclear divisions of the most chalazal megaspore, giving rise to a megagametophyte of the *Polygonum*-type. Callose staining of pollen tubes and tracking of sperm cells within the megagametophyte showed that fertilization occurs 12–14 h after pollination. We also developed a simple whole-mount immunolocalization procedure with sufficient sensitivity to allow the detection of meiotic proteins specifically expressed in the megaspore mother cell. Collectively, the methods developed here for cowpea are likely to be applicable to analyses of gametogenesis in a range of legumes.

## Materials and methods

### Plant material and growth conditions

Seeds of *Vigna unguiculata* IT97K-499-35 and IT86D-1010 were germinated and grown in either a soil mixture of 3:1:1 sunshine soil/vermiculite/perlite (v/v/v), and Osmocote (1.84 kg/m^3^) as a fertilizer, or a mixture of BioGrow (60 % coarse milled composted pine bark >7 mm, 30 % of fine milled composted pine bark 3–6 mm, and 10 % of washed coarse river sand) and Osmocote (1.84 kg/m^3^). Both varieties were grown under greenhouse conditions but did not flower during winter; temperatures in Irapuato were 25–28 °C (spring–summer) and 20–24 °C (autumn–winter), and temperatures in Adelaide were 22–28 °C (summer) and 18–23 °C (winter).

### Cytological analysis

For light microscopy analysis, floral buds were manually dissected and fixed in FAA (ethanol (50 %), formaldehyde (10 %), acetic acid (4 %), and water) for at least 12 h, before being dissected with hypodermic needle and scalpel. Both the bud and the gynoecia length were measured before proceeding to dehydration. Individual gynoecia were dissected under a stereoscope to expose the ovules without detaching them from the placenta. Strings of ovules were progressively dehydrated in ethanol gradients: 15, 25, 40, 50, 60, 75, and 100 % for 30 min each, then a ethanol/methyl salicylate gradient 2:1, 1:1, and 1:2 for 20 min each, and finally mounted on regular microscope slides and 3 coverslips: two on each side of the slide and the third one completely covering the tissue mounted with methyl salicylate 100 %, or directly on well slides. Samples were observed under Nomarski illumination using a DMR Leica or Zeiss microscope. For cytological analysis under confocal microscopy, samples were fixed in FPA (formalin, propionic acid, and ethanol 10:5:7) for at least 12 h, rinsed in l-arginine (12.4 pH), incubated in RNAse for 30 min at 37°, and stained with 0.1 M propidium iodide (pH 12.4) for 1.5 h on ice and in constant agitation. All samples were mounted on conventional microscope slides with *ProLong* or *Vectaschield* (Vector Laboratories, Burlingame USA) and observed on a Zeiss LSM510 META confocal laser scanning microscope with single-track configuration and excitation with a diode-pumped solid-state laser at 568 nm; emission was collected using a band-pass of 575–615.

### Morphometric analysis of the floral bud, gynoecium, and anther

For flower bud size, bracts were removed and the maximum length of the proximal–distal axis was used as a reference for length. The gynoecium was measured following the same axis, from the base of the carpel to the apex of the style. The maximum length of the pre-meiotic primordium was measured from the middle part of the base, where a small protrusion is visible in the placenta, to the apex. The maximum width was taken as the distance across the primordium following the dorsal–ventral axis. The megaspore mother cell (MMC) was measured at its maximal length following both the proximal–distal and dorsal–ventral axis. After meiosis (Stage 4), the maximum length of the meiotic configuration was measured following the proximal–distal axis. The length of the anther was measured from its base to the most distal tip. A total data matrix was generated for all four estimated parameters (gynoecium length, number of dorsal integumentary cells, length of the meiotic configuration, and width of the meiotic configuration), and Pearson correlations were calculated using XLstat.

### Whole-mount protein immunolocalization

Immunolocalization was performed as previously described (Escobar-Guzman et al. 2015), with modifications. Developing ovules were fixed in paraformaldehyde (1 × PBS, 4 % paraformaldehyde, 2 % Triton), under continuous agitation for 2 h on ice, washed three times in 1 × PBS, and embedded in 15 % acrylamide/bisacrylamide (29:1) on pre-charged slides (Fisher Probe-On) treated with poly-l-Lysine as described (Bass et al. 1997). Gynoecia were gently opened to expose ovules by pressing a coverslip on top of the acrylamide. Samples were digested in an enzymatic solution composed of 1 % driselase, 0.5 % cellulase, 1 % pectolyase (all from Sigma) in 1 × PBS for 80 min at 37 °C, subsequently rinsed three times in 1 × PBS, and permeabilized for 2 h in 1X PBS/2 % Triton. Blocking was 1 % BSA (Roche) for 1 h at 37 °C. Slides were then incubated overnight at 4 °C with ASY1 primary antibody used at a dilution of 1:100 (Olmedo-Monfil et al. 2010). Slides were washed for 6 h in 1 × PBS/0.2 % Triton, with refreshing of the solution every 2 h. The samples were then coated overnight at 4 °C with secondary antibody Alexa Fluor 488 (Molecular Probes) at a concentration of 1:300. After washing in 1 × PBS/0.2 % Triton for at least 8 h, the slides were incubated with propidium iodide (PI; 500 µg mL^−1^) in 1 × PBS for 20 min, washed for 30 min in 1 × PBS, and mounted in PROLONG medium (Molecular Probes) overnight at 4 °C. Serial sections on Stage 1 ovules were captured on a laser scanning confocal microscope (Zeiss LSM 510 META), with multitrack configuration for detecting PI (excitation with DPSS laser at 568 nm, emission collected using BP: 575–615 nm) and Alexa 488 (excitation with Argon laser at 488, emission collected using BP: 500–550 nm). Laser intensity and gain were set at similar levels for all experiments. Projections of selected optical sections were generated using ImageJ.

## Results

### Growth, flowering time, and phenology of two cowpea varieties

**Fig. 1:**
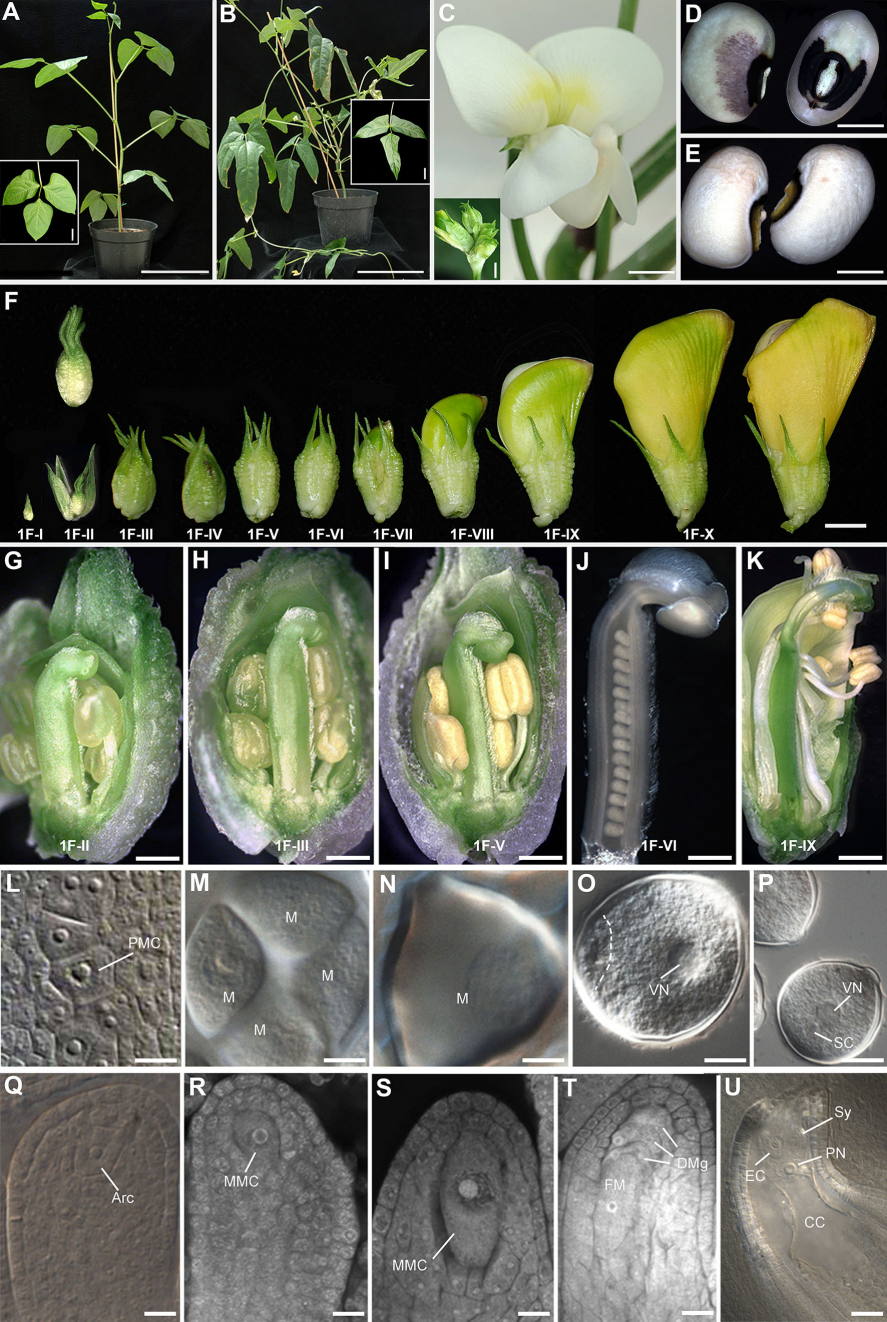
Phenology and reproductive timeline in two varieties of cowpea. Vertically aligned micrographs in **g**–**u** represent corresponding male and female developmental stages within the same floral bud. **a** Vegetative architecture and leaf morphology of IT97K. **b** Vegetative architecture and leaf morphology of IT86D. **c** Mature flower of IT86D; the inset shows floral buds of IT97K. **d** Mature seeds of IT86D. **e** Mature seeds of IT97K. **f** Floral development in IT86D; the numerical register of floral buds corresponds to developmental stages illustrated in **g**–**u**. **g** Dissected floral bud of 2.5 mm in length. **h** Dissected floral bud of 3 mm in length. **i** Dissected floral bud of 4 mm in length. **j** Isolated gynoecia of 3.4 mm in length. **k** Dissected floral bud of 32 mm in length. **l** Differentiated microspore mother cells. **m** A tetrad of microspores. **n** One-nucleate microspore. **o** Two-nucleate microspore. **p** Fully differentiated pollen grain. **q** Ovule primordium containing an archespore. **r** Megaspore mother cell. **s** Megaspore mother cell at the onset of meiosis. **t** Functional and degenerated megaspores. **u** Fully differentiated female gametophyte. *Arc* archespore, *CC* central cell, *DMg* degenerating megaspore; *EC* egg cell, *FM* functional megaspore, *M* microspore, *MMC* megaspore mother cell, *PMC* pollen mother cell, *PN* polar nucleus, *SC* sperm cell, *Sy* synergid, *VN* vegetative nucleus. *Scale bars* **a** and **b** = 13 cm (*inset* = 4 cm); **c** = 5 mm (*inset* = 0.5 mm); **d**, **e** = 4 mm; **f** = 8.5 mm; **g**, **h** = 0.6 mm; **i** = 1.2 mm; **j** = 0.5 mm; **k** = 2.7 mm; **l** = 13 μm; **m**–**o** = 10 μm; **p** = 20 μm; **q**–**s** = 10 μm; **t** = 11.5 μm; **u** = 18 μm

Two transformable *V. unguiculata* varieties, IT86D-1010 and IT97K-499-35 (abbreviated IT86D and IT97K, respectively) with different genetic pedigrees (Supplementary Figure 1) were utilized in this study. Both are photoperiod insensitive, adapted to dry, semiarid regions, and were developed in West Africa. In general, both varieties behave similarly at both growing locations in Irapuato, Mexico, and in Adelaide, Australia, stably expressing clear inter-variety differences in leaf phenology (Fig. 1a, b and insets; see also “Methods”). Contrary to Adelaide, where both varieties had a vine-like habit, in Irapuato, IT86D presented a more vine-like habit with numerous polychotomic ramifications and thin stems, whereas IT97K had dichotomic branching (Fig. 1a, b). Although petals are white with subtle purple during floral development in both IT86D and IT97K, the flowers become yellow at the onset of senescence (Fig. 1c). During the first five (IT86D) to eight (IT97K) weeks after flowering, a variable proportion (50–70 %) of flowers aborted per plant at both locations during the life cycle of both varieties, indicating a variability in the number of flowers that give rise to seeds. At 40–48 h following anthesis and fertilization, the corolla is usually fully detached from the peduncle and only the calyx remains attached at the onset of fruit formation. Non-abortive flowers give rise to pods harboring between 8 and 17 seeds per pod. Seeds of IT86D can be distinguished from those of IT97K by additional purple pigmentation surrounding the hilum or “black-eye” of the seed in IT86D (Fig. 1d, e).

Importantly, flowering time differs in both varieties with flower buds evident in IT86D at 3–4 weeks (Irapuato, Mexico) or 5–6 weeks (Adelaide, Australia) following germination (Fig. 1c), whereas flowering is delayed until 7–8 weeks after germination in IT97K (Fig. 1c, inset). The life cycle of both cowpea varieties is broadly divided into three distinct growth and senescence phases. The first 5 weeks (IT86D) to 8 weeks (IT97K) after germination are characterized by robust vegetative growth, with floral development progressing through the characteristic stages shown in closed (Fig. 1f) and dissected floral buds (Fig. 1g–k). In both varieties, seed yields are maximal during this phase, with approximately 16–17 seeds setting per pod. In the following 6 weeks (IT86D) to 8 weeks (IT97K), the new leaves are significantly reduced in size and floral abortion increases with few pods evident, harboring an average of ten seeds. The third phase is characterized by cessation of leaf and flower formation and seed set, as the plant undergoes senescence.

Male and female gametophyte development begins in developing anthers and ovules of 2.5 mm floral buds in which the anther has reached 0.1 mm and the gynoecium 1.5 mm in length (Fig. 1f–g). All events of pollen and female gametophyte development, progressing from pollen mother cell formation to pollen maturity, and from archespore differentiation to full cellularization of the megagametophyte, respectively, can be correlated with floral bud size and gynoecium length in dissected buds as shown in Fig. 1g–u and Table 1. The elongating stamens grow and curl around the stigma of the gynoecium and dehisce, enabling fertilization when the petals are still closed (Fig. 1k), which partially explains the low degree of outcrossing.

**Table 1:** Reproductive timeline: average floral bud size, average gynoecium length, and gametogenesis in cowpea

Average floral bud size (mm)	Average gynoecium length (mm)	Ovule development; female and male gametogenesis
1	0.5	Ovule primordium initiation; active cell divisions
2.5	1.5	Rapid growth of ovule primordium (80–160 μm in length). Differentiation of subepidermal archespore; differentiation of pollen mother cells in the young anther
3	1.75	Ovule primordium reached 90–210 μm in length; differentiation of the MMC (at least 20 μm in length)
4	2.5	Differentiated MMC has entered prophase I: frequent leptotene and zygotene stages. Initial growth of inner and outer integuments. Ovule has reached a 90° angle with respect to its initial proximal–distal axis. Anther is approximately 200 μm in length; pollen development at the tetrad stage
5	3	Maximum length of MMC is reached (approximately 50 μm). Outer integument is now longer than the inner integument that covers approximately half of the nucellus. Anther approximately 280 μm in length and contains free one-nucleate microspores
5	3.5	Meiosis II has initiated and sometimes has already occurred as four meiotically derived cells are visible. Inner integument covering about half of the nucellus, outer integument has reached the micropylar pole. Rare presence of a two-nucleate stage of female gametophyte
5.5	4	Full differentiation of the functional megaspore, full degeneration of three additional megaspores. Both integuments have reached their full length. Anther is approximately 350 μm in length and contains two-nuclear microspores
5.5–6	4	Two- to four-nucleate stage of female gametophyte development. Three-nucleate pollen often visible
6.5	5–6	Four- to eight-nucleate stage of female gametophyte development. Mature pollen grain
10	7.5–8	Cellularized female gametophyte; sporadic visual identification of rapidly degenerating antipodal cells

### Timeline of male and female gametophytic development

#### Early ovule development

**Fig. 2:**
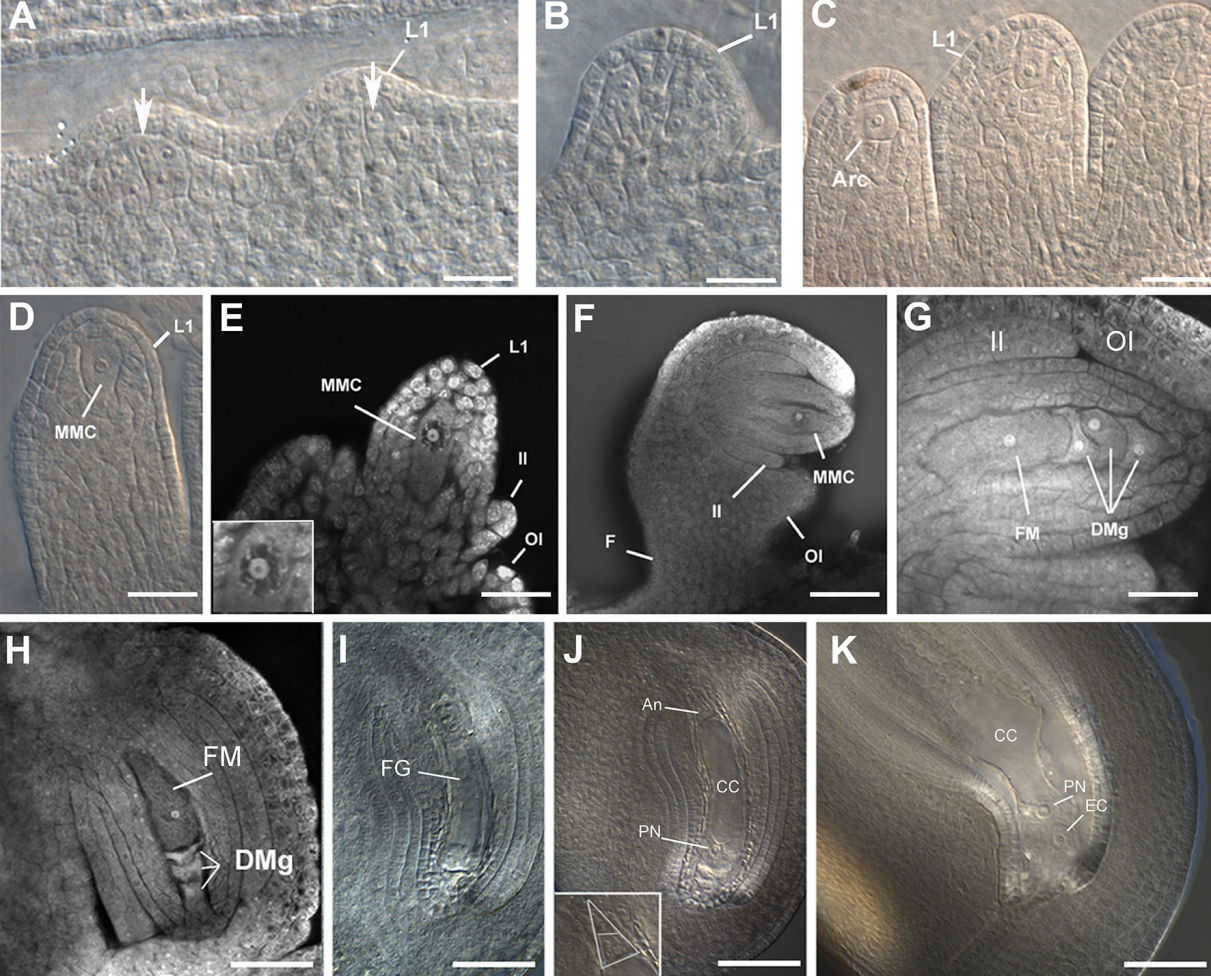
Ovule and female gametophyte development in cowpea. **a** Initial stages of ovule primordium growth; several proliferative cells differentiate in the L2 and L3 layers (*arrows*); the gynoecium is approximately 0.5 mm in length. **b** Ovule primordium prior to archespore differentiation (gynoecium 1 mm). **c** Developing ovules harboring a single archespore (gynoecium 1.5 mm). **d** Developing ovule showing an MMC at the onset of megasporogenesis (gynoecium 1.75 mm). **e** Ovule with an MMC undergoing meiosis I; the nucleus of the MMC is at zygotene stage (gynoecium 2.5 mm). **f** Developing ovule with a fully differentiated MMC (gynoecium 3 mm); the outer integument is longer than the inner integument that covers approximately half of the nucellus. **g** Developing ovule at the end of megasporogenesis. The inner integument has resumed its growth; the functional megaspore (FM) is the meiotically derived cell located at the chalazal pole, adjacent to three degenerating megaspores (gynoecium length 3.5 mm). **h** Developing ovule at the onset of megagametogenesis; a fully differentiated megaspore and degenerated megaspores are within the same cellular plane (gynoecium length 3.75 mm). **i** Developing ovule at the four-nuclear stage of megagametogenesis. **j** Differentiating female gametophyte showing three antipodal cells at the chalazal end (*inset*). **k** Fully differentiated ovule containing a cellularized female gametophyte; the egg cell and polar nucleus are visible. *Arc* archespore, *CC* central cell, *EC* egg cell, *DMg* degenerating megaspore, *F* funiculus, *FG* female gametophyte, *FM* functional megaspore, *II* inner integument, *L1* L1 layer, *MMC* megaspore mother cell, *OI* outer integument, *PN* polar nucleus. *Scale bars* **a** = 18 μm; **b** = 21 μm; **c** = 28 μm; **d** = 23 μm; **e** = 13 μm; **f** = 31 μm; **g** = 12 μm; **h** = 27 μm; **i** = 37 μm; **j** = 12 μm; **k** = 56 μm

Analyses of the first phase of flowering in both IT86D and IT97K varieties showed that the ovules initiate as elongated primordia, emerging anticlinally from internal cell layers of an incipient gynoecium that is approximately 0.5 mm in length from its base to its apex. When the ovule primordium reaches 20–50 μm in length, the L2 and L3 layers exhibit a cluster of differentiated cells having an enlarged nucleus and nucleolus (Fig. 2a, b and Table 1), indicative of active cell division. The first stages of primordium elongation, which occur prior to integumentary initiation, are characterized by a rapid phase of sporophytic ovule cell proliferation. During these stages, the gynoecia grow from 0.5 to 1.5 mm (Figs. 1g, q and 2c; Table 1).

#### Differentiation and division of the megaspore mother cell

When the gynoecium and the ovule reach 1.5 mm and 80–160 μm, respectively, the archespore differentiates at the distal pole of the primordium. The archespore does not abut cells in the L1 epidermal layer, but it is surrounded by subepidermal and possibly L3 cell layers (Fig. 2c). At this same stage, the anther is close to 100 μm in length and contains differentiated pollen mother cells (Figs. 1h, m; Table 1). Apart from elongation, there were no obvious structural differences enabling distinction between the archespore and the MMC, and we therefore arbitrarily classified a cell as an MMC when the archespore enlarged to 25 μm in length. At this time of MMC differentiation, the gynoecium had usually reached 2.5 mm in length and the anther was 200 μm long and contained meiotic tetrads (Figs. 1h, m, r, 2d; Table 1). In the ovule, integumentary growth initiates at the dorsal side of the ovule primordium (Fig. 2d–e). Integumentary growth occurs at stages when the gynoecium length is between 2.5 and 4 mm (Fig. 1h–j).

The maximum length of the MMC (approximately 50 μm) is usually reached in floral buds containing gynoecia that are 3 mm in length (Figs. 1i, s, 2f), and when 200 μm long anthers contain uninucleate microspores (Fig. 1i, n). In the ovule, the inner integument has surrounded about half of the nucellus and the outer integument is still shorter than the inner integument (Fig. 2f). When the gynoecium is approximately 3.5 mm in length, the MMC undergoes or finishes meiosis II, as four meiotically derived cells are visible (Fig. 2g). The inner integument arrests growth at this stage and does not surround the base of the embryo sac or contribute to the micropyle (Fig. 2h).

Meiosis occurs in the same cellular plane, giving rise to a tetrad of megaspores. Contrary to previous reports, the functional and surviving megaspore is not of micropylar location as is characteristic of developing Oenothera-type embryo sacs, but is located at the most chalazal pole of the post-meiotic tetrad. Furthermore, it undergoes three rounds of mitosis, indicating that cowpea undergoes the classic Polygonum-type of megagametogenesis (Fig. 2g). In 4 mm long gynoecia, megasporogenesis usually has concluded and the functional megaspore is differentiated (Fig. 1j, t). The anthers are close to 350 μm in length at this stage, and microspores have divided, characteristic of the two-nuclear stage (Fig. 1j, o). The outer integument has largely completed formation of the micropyle (Fig. 2g, h), and a vascular bundle has differentiated in the funiculus, terminating in the chalazal region of the ovule.

#### Megagametogenesis

Megagametogenesis occurs rapidly after the functional megaspore is around 80 μm in length along its longitudinal axis (Fig. 2h). Once the gynoecium is 5 mm in length, the female gametophyte is generally at the two- to four-nuclear (2NFG and 4NFG) stage (Fig. 1k, u), and the anthers contain tricellular pollen grains (1K and 1P). The female gametophyte undergoes cellularization rapidly after eight nuclei form in the gynoecia between 5 and 8 mm in length (Fig. 2i). Antipodal cells are transiently evident at the chalazal pole of the embryo sac confirming formation of a Polygonum-type embryo sac, but degrade soon after cellularization (Fig. 2j). The egg cell has a tear-like shape and contains a conspicuous nucleus with prominent nucleolus usually located at its chalazal pole, contrary to the synergids in which the nucleus is barely visible (Figs. 1u, 2j, k). At the onset of self-pollination, ovules contain fully differentiated female gametophytes, and a voluminous, highly vacuolated central cell in which the two polar nuclei have fused, forming a primary endosperm nucleus located adjacent to the egg apparatus (Fig. 2k).

### Dynamics of pollen tube growth and double fertilization

**Fig. 3:**
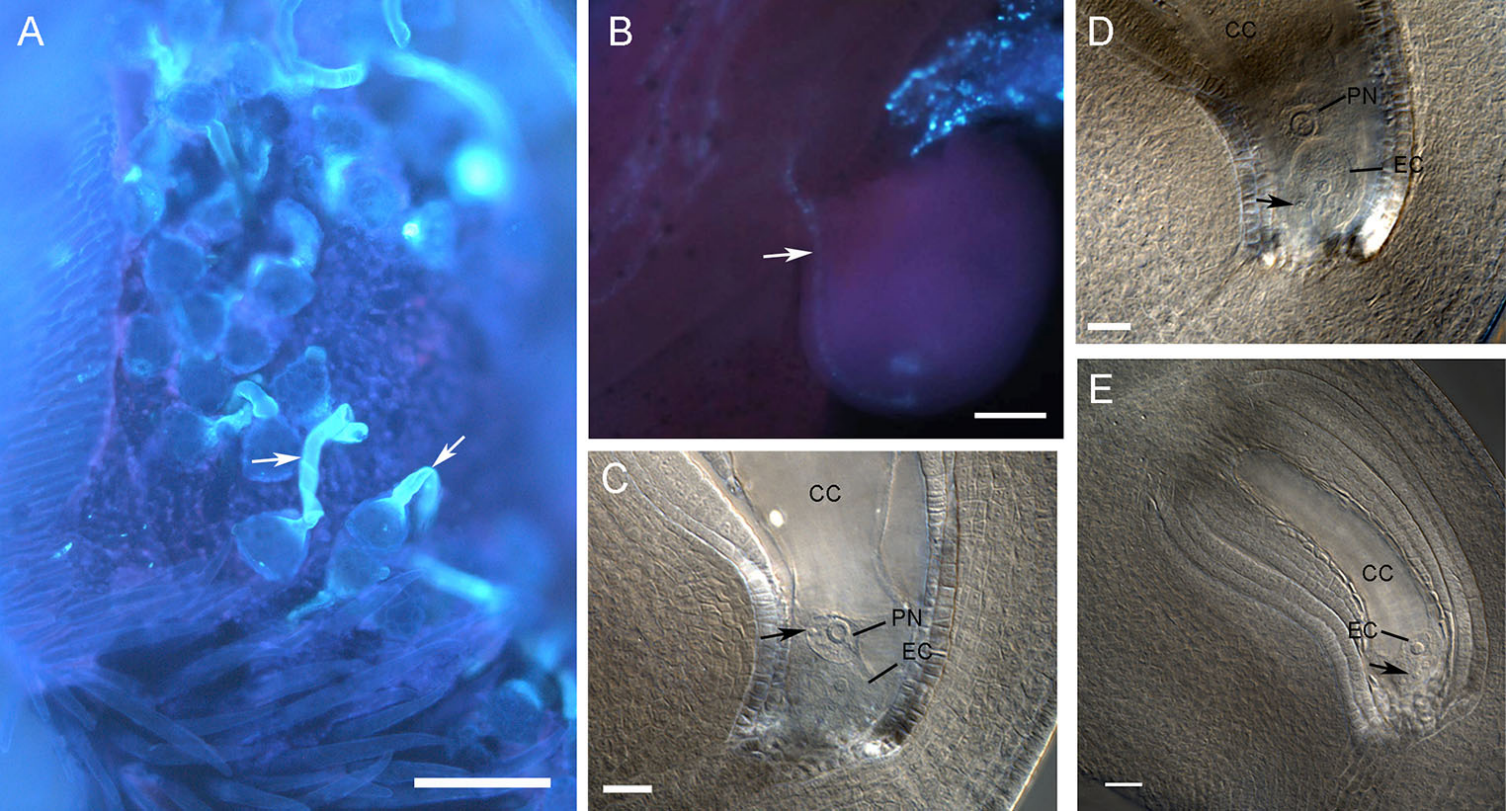
Dynamics of pollen tube growth and double fertilization in cowpea. **a** Stigma showing pollen tube germination (*arrows*) 3 h after cross-pollination. **b** Pollen tube (*arrow*) having reached the micropyle of a fully differentiated ovule. **c** Fertilization of the central cell; a sperm cell nucleus (*arrow*) is visible within the primary endosperm polar nucleus; the egg cell has acquired a zygotic-like morphology. **d** Fertilization of the egg cell; a sperm cell (*arrow*) is adjacent to the egg cell membrane (*arrowhead*). **e** Fertilization of the egg cell; a sperm cell nucleus (*arrow*) is visible within the egg cell cytoplasm. *CC* central cell, *EC* egg cell, *PN* polar nucleus. *Scale bars* **a** = 40 μm; **b** = 50 μm; **c**–**e** = 20 μm

We conducted manual cross-pollinations with flowers of IT97K, collecting and fixing gynoecia at consecutive time intervals following pollination, and observing pollen tube growth using aniline blue staining to establish the time of fertilization. Typically, 50–70 % of flowers aborted 24–48 h after pollination (HAP) without initiating viable seed development. We estimated the distance from the surface of the stigma to the base of the funiculus of the most apical ovule to be 1.4 cm in length in cleared whole gynoecia. Pollen grains usually germinated within 3–4 HAP following cross-pollination (Fig. 3a; Table 2). At 6 HAP, less than 10 % of germinated pollen tubes were within the apical region of the style, and by 12 HAP, the same proportion had reached the funiculus of the most apical ovule, suggesting that rapid pollen tube growth occurs between 6 and 12 HAP (Fig. 3b; Table 2). Sperm cell delivery was observed within a degenerating synergid between 12 and 14 HAP. Although it was not possible to consistently visualize a degenerated synergid in whole-mount cleared specimens, the sperm nucleus could be clearly identified within the primary endosperm nucleus of the central cell, or in the cytoplasm or nucleus of the egg cell (Fig. 3c–e). Following fertilization, the egg cell acquires a spherical morphology reminiscent of zygotes in many previously characterized legume species.

**Table 2:** Dynamics of pollen tube growth in cross-pollinated flowers of cowpea

Time after pollination (h)	Observed styles	Pollen tube length	Comments
1	15	Not germinated	
3	15	50–100 μm	The pollen tubes have germinated but have not penetrated the stigma
4	12	90–210 μm	Less than 10 % of germinated pollen tubes penetrating the style
6	13	300–350 μm	Less than 10 % of germinated pollen tubes penetrating the style
12	10	1.2–1.4 mm	All ovules in the gynoecia showed the presence of a pollen tube at the surface of the funiculus

### Sporophytic parameters such as gynoecium length and integument growth can be used to predict stages of megasporogenesis

**Fig. 4:**
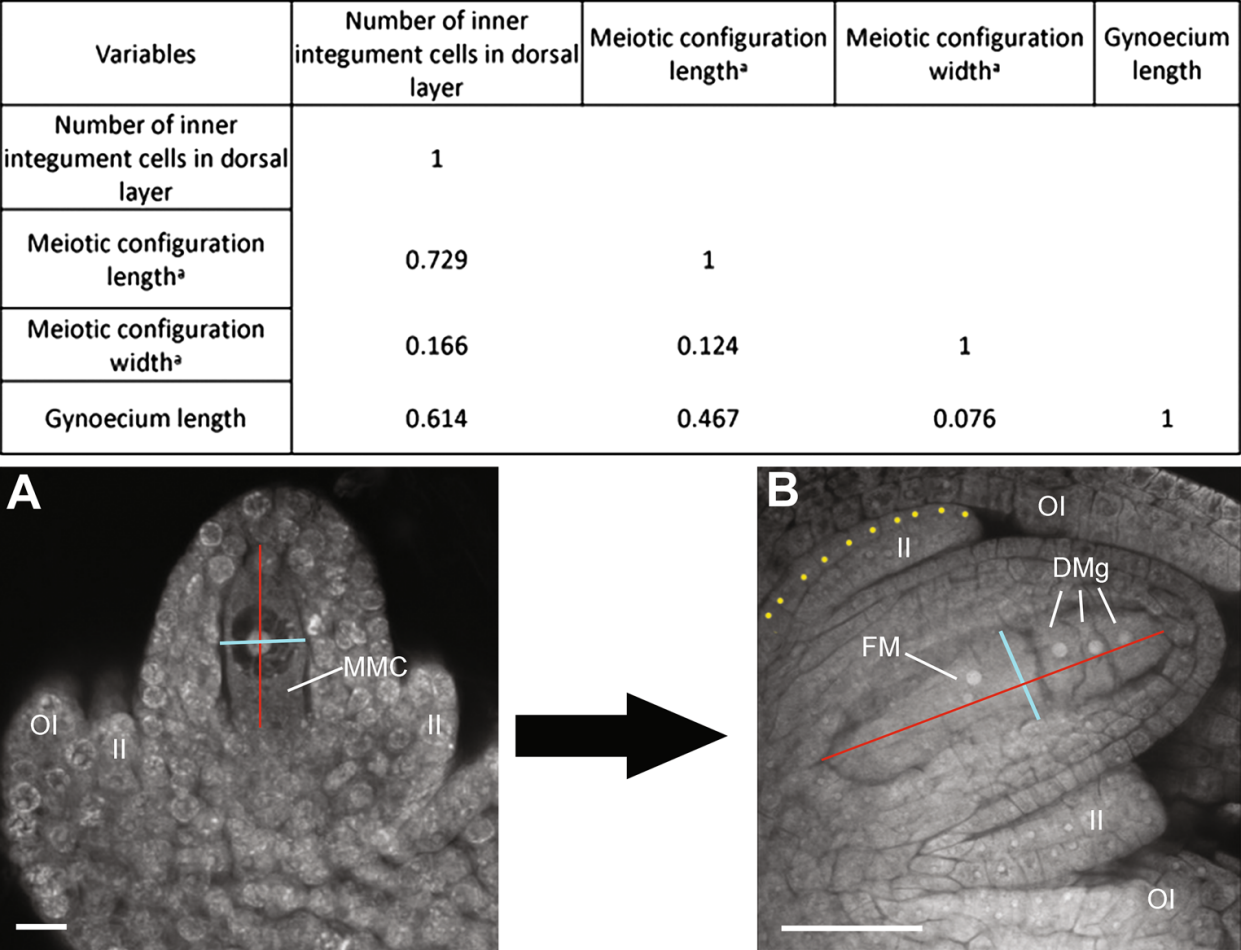
Correlations between sporophytic and gametophytic parameters in ovules undergoing megasporogenesis. The length of the gynoecium, the number of inner integument cell layers (*yellow* dots in *B*), and the length and width of the meiotic configuration were scored in developing ovules that were staged between the fully differentiated MMC and fully differentiated functional megaspore. *DMg* degenerating megaspore, *FM* functional megaspore, *MMC* megaspore mother cell, *II* inner integument, *OI* outer integument. *Scale bars* *A* = 10 μm; *B* = 20 μm. ^a^The distance measured for the meiotic configuration length (*red*) and width (*light blue*) is highlighted in *A* and *B*

To assess the possibility of correlating sporophytic and gametophytic parameters during early ovule development, we measured morphological characteristics of the gynoecium and the inner integument relative to the megasporogenic stage, encompassing the full differentiation of the MMC to the end of meiosis and differentiation of the functional megaspore (*n* = 296). During this developmental timeframe, the minimum and maximum length of a gynoecium was 0.5 and 4 mm, respectively. For the inner integument, we measured the maximum number of longitudinal cell layers that were found at the dorsal pole of the ovule; the maximum and minimum number of cell layers score was 4 and 21, respectively. The analysis of female meiotic configurations included stages encompassing megasporogenesis; in some cases, it only included the MMC, and in others the four meiotically derived megaspores at different stages of development or degeneration (Fig. 4). In general, the meiotic configuration width was poorly correlated with the three other parameters, suggesting that the volume of the MMC and the megaspores along the dorsal–ventral axis is highly variable. In contrast, the length of the meiotic configuration was positively correlated with the sporophytic parameters; the best correlation (0.73) was obtained with the number of inner integument cells, suggesting that the two developmental programs are interrelated and at least partially synchronized. In addition, the number of inner integument cells was also positively correlated with the length of the gynoecium, confirming that the latter can be used as an approximation to predict the developmental stage of the female gametophyte.

### Immunolocalization of meiotic proteins in the ovule

**Fig. 5:**
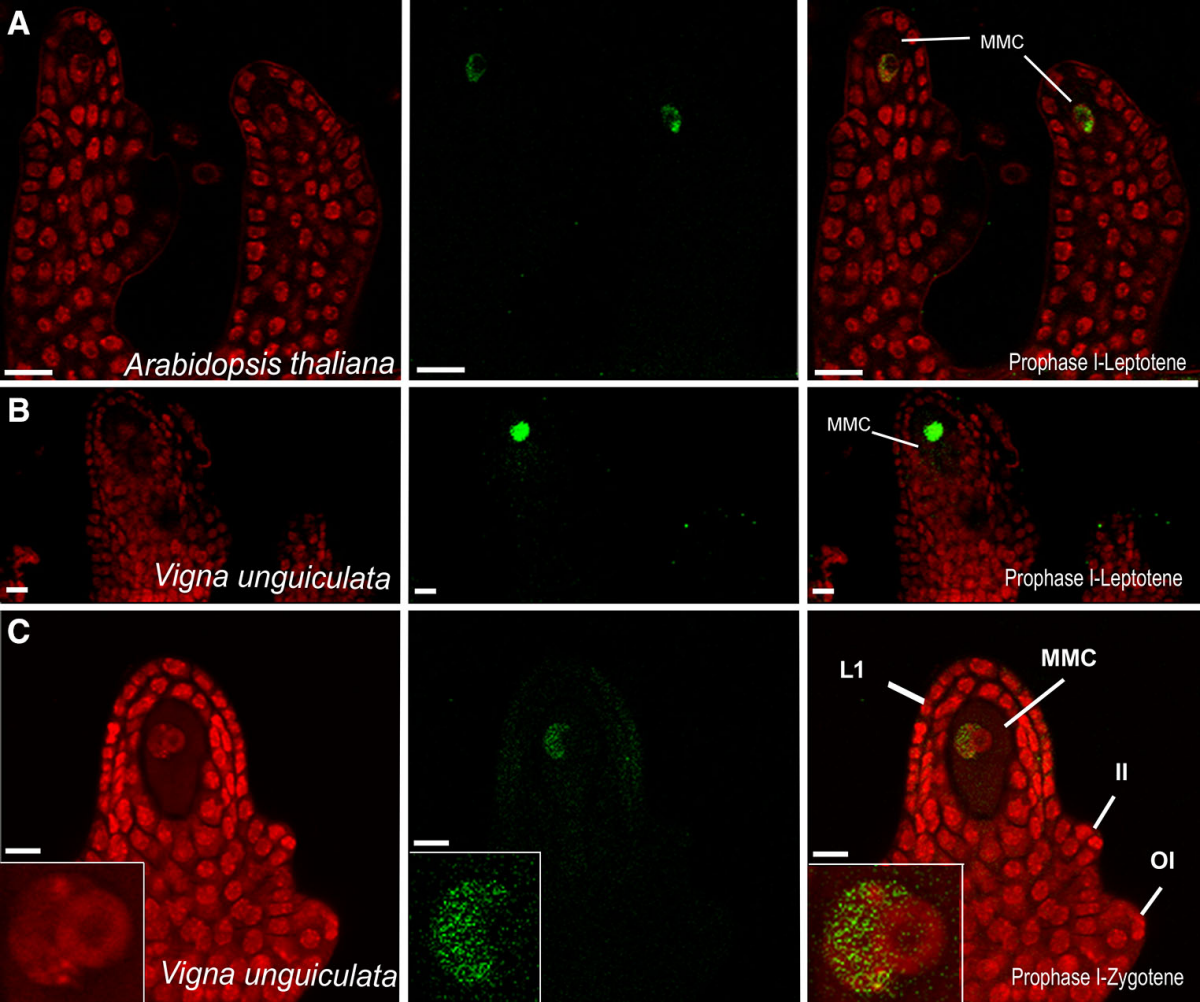
Whole-mount immunolocalization of ASY1 in the developing ovule of *Arabidopsis thaliana* and cowpea IT86D. A polyclonal antibody against the maize protein ASY1 (*green*) was used in whole-mount immunolocalization; cells were counter-stained with propidium iodide (*red*). **a** Ovules of Arabidopsis showing ASY1 localization in the MMC. **b** Ovule of cowpea showing ASY1 localization in the MMC. **c** Ovule of cowpea showing an MMC at prophase I of meiosis; MMC chromatin is condensed at one of the edges of a conspicuous nucleolus that presents a large nucleolar vacuole. *L1* L1 layer, *MMC* megaspore mother cell, *II* inner integument, *OI* outer integument. *Scale bars* **a**–**c** = 10 μm

Assessing any heterochronic divergence in developmental traits related to such a highly dynamic process as megasporogenesis requires novel tools to rapidly determine protein localization in the ovule. Meiosis is of particular significance for manipulation for the purpose of generating clonal seed. Therefore, we attempted the adaption in cowpea of a whole-mount immunolocalization procedure initially developed to follow the subcellular localization of proteins during female meiosis in *Arabidopsis thaliana* (Escobar-Guzman et al. 2015). In Arabidopsis, the procedure can be used to follow meiotic proteins during megasporogenesis, allowing the analysis of structural details such as the formation of the axial element of the synaptonemal complex on the basis of an antibody raised against ASY1 (Fig. 5). The protocol is based on implemented procedures used to observe structural features in chromosomes of meiocytes of maize (Bass et al. 1997) and the function of epigenetic pathways in Arabidopsis ovules (Olmedo-Monfil et al. 2010). To adapt the protocol to the crassinucellate ovule of cowpea, we performed serial experiments using the same antibody that was used for Arabidopsis and maize, but increasing the time of exposure to an enzymatic cocktail composed of driselase, pectolyase, and cellulase. Whereas no signal was detected in ovules harboring an early archespore (data not shown), consistent ASY1 localization was restricted to the MMC at initial stages of megasporogenesis (Fig. 5). Initially localized throughout the MMC nucleus, the signal was restricted to one of the dorsal–ventral poles of the MMC nucleus at prophase I. Counterstaining with propidium iodide showed that no signal was present in a large MMC nucleolus containing a nucleolar vacuole. These results suggest that the signal indeed corresponds to the subcellular localization of ASY1 at initial stages of megasporogenesis, raising the possibility of using the procedure to compare the dynamics of male and female meiosis in cowpea, and examining protein localization at other stages of female reproductive development.

## Discussion

The development and use of self-perpetuating improved hybrids of legume crops cultivated by smallholder farmers of sub-Saharan Africa and Southern Asia could substantially transform subsistence agriculture by affordably offering superior germplasm and new sources of revenue on the basis of seeds adapted to local or regional conditions. We hypothesize that if sexual reproduction of a hybrid could be altered such that the offspring are identical to the parental hybrid plant, heterosis would be preserved in harvested seeds. This could be achieved if gametes were formed only by mitosis, viable endosperm was formed, and the embryo in the seed retained the hybrid genotype, mimicking apomixis, or asexual seed formation. We are attempting to develop self-reproducing hybrids in cowpea, based, in part, on a recent combination of Arabidopsis mutants that have provided proof-of-concept of clonal seed formation (Marimuthu et al. 2011; Hand and Koltunow 2014; Gilbert 2015). The implementation of this type of technology requires new knowledge and experimental tools that depend on a detailed understanding of the genetic basis and molecular mechanisms that control reproductive development.

We have initiated a series of developmental studies that take advantage of cowpea local varieties to conduct a detailed cytological characterization of ovule and female gametophyte formation, identify correlation values between some sporophytic and gametophytic parameters, and establish whole-mount immunocytological procedures that allow a fast and reliable assessment of protein expression in the ovule. As in other species of legume investigated to date, the ovule of cowpea is anatropous, bitegmic, and crassinucellate. But contrary to other legume species in which initial stages of primordium initiation are characterized by homogenous states of cell differentiation (Mitchel 1975; Kennel and Horner 1985), the pre-meiotic ovule of cowpea is characterized by a cluster of up to 20 differentiated L2 and L3 cells that are present immediately following the anticlinal divisions that give rise to the incipient ovule bulge, but absent at later stages of elongation, i.e., before differentiation of the archespore. Although the developmental nature of these cells remains to be elucidated, their location and ephemeral clustered organization suggests that they represent a proliferative stage of rapid cell divisions that give rise to the pre-meiotic primordium.

A single archespore differentiates in the ovule of cowpea. As in some previously characterized legumes, but contrary to most angiosperms, the MMC is separated from the L1 by one additional sporophytic cell layer. Brown (1917) was first to report this unusual position for the female gamete precursor. In the case of *P. vulgaris*, a careful analysis of cell divisions showed that the MMC origin was in the L2 layer, but that an unusual pattern of divisions at the apex of the primordium gave rise to a second layer of cells separating the MMC from the L1 (Brown 1917). Although not discussed by the authors, our own interpretation of data presented by Kennel and Horner (1985) indicates that the MMC in *Glycine max* is also separated from the L1 layer by one or two sporophytic cell layers. In *Vicia faba*, Mitchel (1975) reports that a multicellular archeosporium differentiates beneath two layers of nucellar cells. Contrary to *P. vulgaris* and cowpea, multiple archespores differentiate in *V. faba*, as is also the case in *V. americana*, *V. dasycarpa*, and V. *villosa* (Rembert, 1969). In the case of *V. faba* as in *G. max*, the cytological characterization is completely based on the analysis of sectioned samples, complicating a possible determination of the patterns of cell division that occur in the L2 layer. Although a definitive elucidation of the MMC cell linage will require further cytological and genetic analysis, on the basis of the classic study by Brown (1917) we propose the hypothesis that the female gametic lineage in cowpea is of L2 origin, but that subsequent particularities of L2 layer divisions determine the final location of the MMC. Our results also suggest that the type of megagametogenesis that prevails in cowpea should be reconsidered. In the case of cowpea, a single published report describing ovule and female gametophyte development on the basis of thick paraffin-embedded sections concluded that the variety “Adzuki” followed an Oenothera-type of megagametogenesis where the micropylar-most megaspore initiates mitotic embryo sac formation and two rounds of mitosis are observed (Ojehomon and Samyaolu 1970). This is contrary to most Faboideae species in which the most chalazal megaspore undergoes three rounds of mitosis to develop into a Polygonum-type of female gametophyte (Rembert 1969). Other exceptions include *Trifolium repens* and *Vicia faba*, in which an epichalazal megaspore exceptionally can give rise to the functional megaspore (Mitchel 1975; Rembert 1977), as well as *Laburnum anagyroides* and *Pogamia*, in which the Allium-type of female gametogenesis was reported (Rembert 1966). Our results show that cowpea invariably forms a linear tetrad in which the surviving megaspore is located at the chalazal pole of the nucellus, indicating that the female gametophyte is indeed monosporic but of the Polygonum-type, which is the case for most species of the Faboideae reported to date. This observation was confirmed by the presence of three antipodals at the chalazal pole of the differentiating female gametophyte. At maturity, when the egg apparatus is differentiated, the antipodals have degenerated.

Our combination of cytological and morphometric analyses has allowed the establishment of a female reproductive calendar that can be used to anticipate a range of gametophytic stages on the basis of a sporophytic timeline that can be followed with help of specific parameters such as bud size or gynoecium length. We propose that these parameters could be particularly useful to identify and estimate potential heterochronic events in comparison with wild-type gametophytic development in cowpea. Several studies have indicated that changes in cell specification and method of reproduction caused by natural mechanisms, such as diplospory or apospory, or by specific mutants affecting megasporogenesis can cause important differences in the timing of female meiosis or megaspore differentiation (Sharbel et al. 2010; Carman et al. 2011; Rodriguez-Leal et al. 2015). Although our analysis did not provide a significantly high positive correlation between sporophytic and gametophytic parameters, the number of cell layers present in the developing dorsal inner integument could provide a useful approximation. As is the case of most flowering species in which the correspondence between male and female gametogenesis has been investigated, in cowpea the pollen grain differentiates more rapidly than the female gametophyte. Ovule primordia harboring an archespore were present in flowers that contained anthers in which the pollen mother cell (PMC) was at the onset of meiosis; however, tetrads of microsporocytes were present in flowers in which the ovules contained a MMC at the onset of meiosis, indicating that male meiosis occurs more rapidly than female meiosis. The slower progression of meiosis in the female was corroborated by the presence of highly vacuolated uninucleate microspores at stages in which the MMC had not yet divided. At the onset of megagametogenesis, when the ovule exhibits a large functional megaspore, pollen grains contain a large vegetative cell and a small sperm cell, confirming that the first mitotic division of microgametogenesis occurs before the initiation of megagametogenesis. As expected, the fully differentiated pollen grain of cowpea contains one vegetative and two sperm cells before germination. Despite the equivalency in the time of differentiation of the MMC and the PMC within a flower, the developmental difference in timing is prevalent during both sporogenesis and gametogenesis, suggesting that male meiosis occurs more rapidly than female meiosis.

We also show that the adaptation of a previously published whole-mount immunolocalization procedure allows detection of proteins localized in single cells of the developing ovule, in particular the MMC. By contrast to procedures that require sectioning or smearing to meiocytes from sporophytic cells, in cowpea the procedure preserves the constitution of the ovule, providing a temporal and spatial context to the meiotic division, and opening the possibility for comparing the dynamics of protein localization during female and male meiosis. We anticipate that the procedure will be useful for the analysis of developmental pathways related to the control of archespore differentiation, the characterization of mutants that affect megasporogenesis and require quantification of female meiotic defects within the same gynoecium, or the observation of female meiosis in mutants that eventually lead to apospory or the formation of unreduced gametes.

The developmental timeline, gametogenic description, and experimental tools presented here provide a necessary framework to support large-scale genomic efforts as a source of information that could support marker-assisted selection, laser-capture microdissection, transcriptional profiling, traditional breeding, and genetic engineering for the production of improved varieties of cowpea. Fundamental genome resources including large transcript datasets from specific cell types, genetic and physical maps, and genomic assemblies promise to establish the convergent technological support necessary to develop improved hybrids. We propose that the framework of cytological observations and experimental tools here described could also provide technical adaptations that could soon allow the analysis of reproductive alternatives as a first step to attempt the manipulation of sexual reproduction in cowpea for yield improvement.

### **Author contribution statement**

 AMK and JPVC conceived and designed the research. RSG, SJ, and NSL conducted the experiments, analyzed the data, and participated in manuscript preparation and writing. AMK and JPVC wrote the manuscript.

## Electronic supplementary material

Below is the link to the electronic supplementary material.

Supplementary material 1 (DOC 107 kb)
