# Supplementary figures and images for: New observations on gametogenic development and reproductive experimental tools to support seed yield improvement in cowpea [Vigna unguiculata (L.) Walp.]
Source: Plant Reprod. 2016 Jan 4;29:165–77. doi: 10.1007/s00497-015-0273-3 (PMC4909796; doi:10.1007/s00497-015-0273-3)

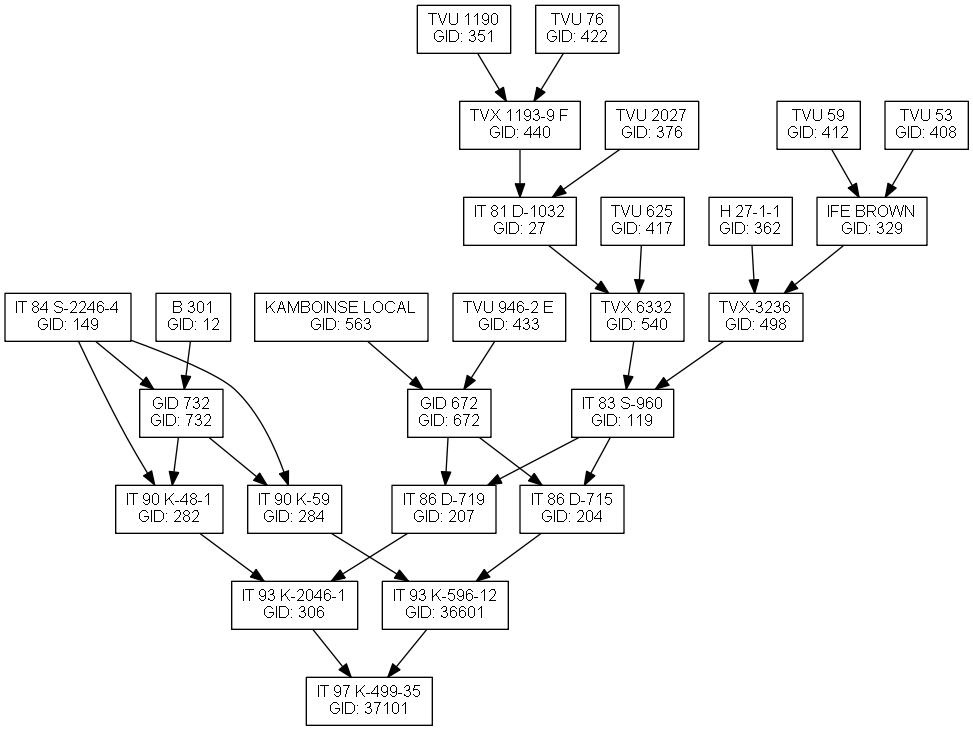


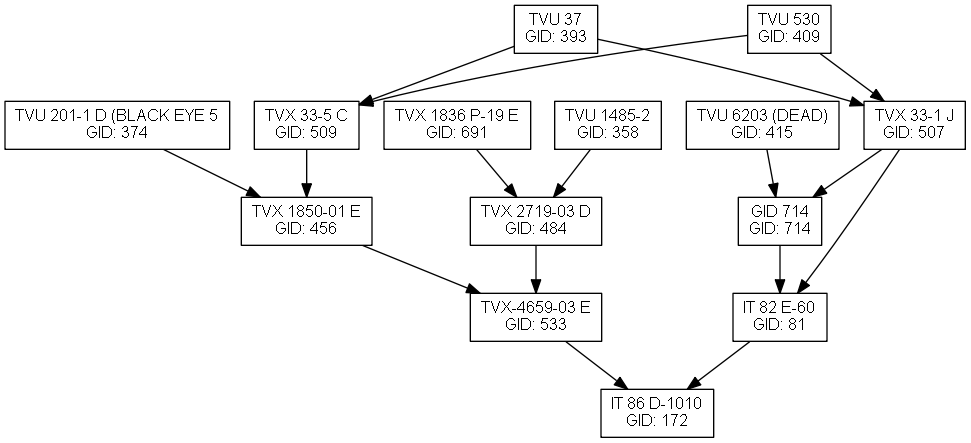


**Supplementary Figure 1. Pedigrees of cowpea IT86D-1010 and IT97K-499-35.**

Supplement: Supplementary file 1 — Supplementary material 1 (DOC 107 kb) [file 497_2015_273_MOESM1_ESM.doc]
